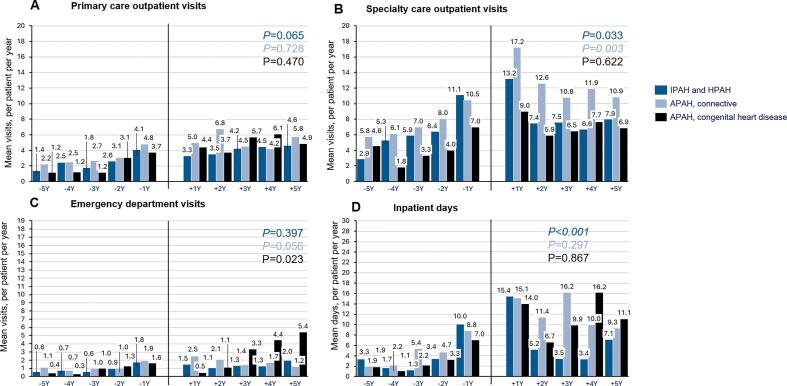# Economic burden of pulmonary arterial hypertension (PAH) and chronic thromboembolic pulmonary hypertension (CTEPH) in Finland

**DOI:** 10.1016/j.ijcha.2024.101534

**Published:** 2024-10-22

**Authors:** Markku Pentikäinen, Piia Simonen, Pauliina Leskelä, Terttu Harju, Pertti Jääskeläinen, Christina Wennerström, Nikolaj Bødker, Eija Heikkilä, Mari Lahelma, Riikka-Leena Leskelä, Airi Puhakka, on behalf of the FINPAH study group, Elina Heliövaara, Katriina Kahlos, Pentti Korhonen, Tiina Kyllönen, Kirsi Majamaa-Voltti, Anu Turpeinen, Helena Tuunanen, Ville Vepsäläinen, Tapani Vihinen

**Affiliations:** a Heart and Lung Center, Helsinki University Hospital, Helsinki, Finland; b University of Helsinki, Helsinki, Finland; c Tampere University Hospital, Tampere, Finland; d Oulu University Hospital, Oulu, Finland; e Kuopio University Hospital, Kuopio, Finland; f Janssen-Cilag AB, Solna, Sweden; g Janssen-Cilag A/S, Birkerød, Denmark; h NHG Finland, Nordic Healthcare Group, Helsinki, Finland; i Janssen-Cilag Oy, Espoo, Finland; j Turku University Hospital, Turku, Finland; k Satasairaala Hospital, Pori, Finland

**Keywords:** Pulmonary arterial hypertension, Chronic thromboembolic pulmonary hypertension, Healthcare resource use, Healthcare cost

## Abstract

**Objectives:**

Given that pulmonary arterial hypertension (PAH) and chronic thromboembolic hypertension (CTEPH) are rare yet severe subtypes of pulmonary hypertension significantly impacting patients’ lives, this study analyzed the total societal costs of these conditions in Finland.

**Methods:**

PAH (n = 247) and CTEPH (n = 177) patients diagnosed between 2008 and 2019 were analyzed for primary and specialty outpatient visits, emergency visits, hospitalizations, home and institutional care, sick leaves, disability pensions, and drug costs for 5 years before and after diagnosis.

**Results:**

In PAH and CTEPH, annual specialty care number of outpatient visits increased from 3.8 and 3.3 (5 years before diagnosis) to 13.8 and 9.5 one-year post-diagnosis, then decreased to 9.2 and 4.0 at 5 years post-diagnosis. Annual inpatient days rose from 2.8 and 2.7 to 16.1 and 19.7 pre-diagnosis, then fell to 10.2 and 3.5 post-diagnosis, respectively. Within 5 years post-diagnosis, in working-age 70 % PAH and 42 % CTEPH patients received disability pensions. Drug therapy accounted for most costs (67 % in PAH and 60 % in CTEPH), followed by inpatient care, disability pensions, and outpatient care. Total costs were significantly lower for CTEPH, especially after pulmonary endarterectomy. Among PAH subtypes, the highest costs were in patients with PAH associated with connective tissue diseases.

**Conclusions:**

PAH and CTEPH cause a significant economic burden on patients and society with considerable differences depending on the PAH subtype and whether the patient has undergone PEA operation or not.

## Highlights

• PAH and CTEPH cause a substantial economic burden on patients and society, with costs starting to increase years prior to diagnosis.
• Care costs for PAH were significantly higher than for CTEPH over the five years post-diagnosis.
• For CTEPH patients, those who underwent PEA surgery had significantly lower overall costs compared to non-operated patients.
• 70% of working-age PAH patients were on disability pension five years post- diagnosis, indicating significant indirect costs and societal impacts.

## 1 Introduction

Pulmonary arterial hypertension (PAH) is a severe form of pre-capillary pulmonary hypertension characterized by functional and structural changes in small pulmonary arteries [1]. Chronic thromboembolic pulmonary hypertension (CTEPH) is a rare complication of acute pulmonary embolism (PE) [2]. Both conditions are associated with a poor prognosis. The five-year survival rate for PAH varies by baseline risk and treatment, estimated between 32 and 76 % [3]. For inoperable CTEPH, the 5-yr survival is around 70 % but can be improved up to 90 % with PEA, BPA and medical therapy or their combination [4]. The diagnosis of both PAH and CTEPH can often be delayed due to nonspecific symptoms like dyspnea, delaying timely treatment initiation [5].

Both PAH and CTEPH cause substantial economic burden. Several studies have shown increased healthcare resource use (HCRU) and costs associated with PAH and CTEPH post-diagnosis [6], [7], [8], [9], [10]. Additionally, elevated HCRU has been observed before diagnosis: In Sweden, HCRU for PAH [9] and CTEPH patients [8] was significantly higher three and five years before diagnosis, respectively, compared to a non-diseased control group. Moreover, PAH and CTEPH patients experience greater productivity loss, including increased sick leaves and disability pensions post-diagnosis. Furthermore, this productivity loss exceeds that of the control group even five years before diagnosis [8], [9].

The aim of this study was to analyze the societal cost of PAH and CTEPH patients 5 years before and after the diagnosis in Finland taking into account primary and specialty care, home and institutional care, sick leaves, disability pensions and actual drug costs based on purchases. Additionally, the costs were analyzed between PAH subtypes and between operated and non-operated CTEPH patients.

## 2 Material and methods

### 2.1 Study design

This study is a retrospective, non-interventional database study based on individual-level data from Finnish national registers and considers the years 2003–2021. The relatively long study period ensures that all study participants, described below, have at least five years history data prior to first diagnosis of PAH/CTEPH and follow-up data up to five years. Study participants were followed from 5 years before index date to 5 years after index date or until censoring (time of death or lost to follow‐up at the end of study period), whichever occurred first. The individual index date was defined as the date of diagnosis (date of diagnostic right heart catheterization).

Individual-level follow-up and history data related to HCRU, drug utilization, comorbidities, cost, and productivity loss were collected from several data sources (Supplementary Table 1). Information about PAH subtypes, date of PAH or CTEPH diagnosis, hospital medications, procedures, and deaths during 1.1.2008–31.12.2020 was collected in the previous FINPAH-study [11], [12].

### 2.2 Study population

The study population consisted of patients diagnosed with PAH or CTEPH in Finland during 1.1.2008–31.12.2019. This study utilized patient cohort already identified in the FINPAH1 study [11], [12], where the following inclusion criteria was used: patients at least 18 years of age with.

i. PAH (ICD-10 code I27.0, I27.8, I27.9, Q21.8) or
ii. CTEPH (ICD-10 code I27.2) or
iii. use of PAH- or CTEPH-specific drugs (ATC B01AC11: Iloprost, B01AC21: Treprostinil, B01AC27: Selexipag, C02KX01: Bosentan, C02KX02: Ambrisentan, C02KX03: Sitaxentan, C02KX04: Macitentan, C02KX05: Riociguat, C02KX52: Ambrisentan and Tadalafil, G04BE03: Sildenafil for PAH or CTEPH, G04BE08: Tadalafil for PAH or CTEPH) or calcium channel blockers (ATC C08) or
iv. symptoms obviously indicating PH (with later verification of PAH or CTEPH), recorded at least once in 2008–2019.

The patients with Eisenmenger syndrome or univentricular heart (UVH)/Fontan circulation were excluded.

### 2.3 Study outcomes

Annualized healthcare use (HCRU) of PAH and CTEPH patients was assessed in public primary healthcare, specialty healthcare, and social care. In primary and specialty care, outpatient visits, emergency department (ED) visits as well as inpatient days were analyzed. Primary healthcare included contacts in home care. Social care included institutional care and rehabilitation episodes.

Drug utilization was analyzed based on medicines purchased from pharmacies which included all outpatient medication purchases with doctor’s prescriptions.

Cost estimates included both direct and indirect costs. Direct costs associated with HCRU were estimated using a bottom-up approach, where each event type was assigned a unit cost (i.e., cost per primary care outpatient visit, cost per specialty care inpatient day) and the unit cost was multiplied by the number of units used. The unit cost reflects the average cost of visits or inpatient days include e.g. personnel, materials, operative/invasive treatments, and facilities. Unit costs for both social and health care resource use were derived from the publication by the National Institute for Health and Welfare [13]. Profession and/or medical specialty were considered in the unit costs. Unit costs from 2021 were used for all years. Cost for outpatient prescription drugs were directly available in the dataset. Indirect costs included sick leaves, and disability pensions of PAH and CTEPH patients. We used the actual paid reimbursements and pensions as they were available in the dataset. The sick leave reimbursement register contains only sick leaves exceeding 10 days, and therefore the wage for the 10 first days (paid by employer) was valued based on the mean gross salary in Finland 2021 from Statistics Finland. All costs derived directly from registers were adjusted to 2021 prices using Price index of public expenditure [14].

Annual mean HCRU 5 years before and 5 years after index date (outpatient visits, ED visits, inpatient stays, drug utilization) and related costs per patient were calculated using complete cases approach, thus, including patients uncensored and alive at the end of each year. The total 5-year mean costs from pre‐ and post‐index date were calculated using all available samples adjusted for censoring using the Zhao and Tian estimator [15]. The estimator accounts for censoring by weighting uncensored costs by the likelihood of being censored at the time of event, as well as using cost history from both censored and fully observed cases.

### 2.4 Statistical analysis

Data are described as mean and standard deviation (SD) or median and interquartile range (IQR) for continuous variables, and frequency (n) and proportions (%) for categorical variables. The overall population-level longitudinal trend in HCRU parameters and costs trend over the years after diagnosis (years + 1 to + 5) was analyzed using linear regression. Differences between the 5-year total costs pre- vs. post-index date were tested using t‐test. All analyses were performed using R software (version 4.0.4). A two-tailed test of significance was assumed, and the alpha level was set a-priori at 0.05.

### 2.5 Ethical consideration

The study followed good clinical practice following the declaration of Helsinki and the laws and regulations applicable in Finland. This retrospective register study does not require informed consent under Finnish legislation. There is no additional risk to participants, as data will be collected from existing medical records and patients will not be directly involved. Researchers had access only to pseudonymized data.

## 3 Results

### 3.1 Study population

A total of 424 patients were diagnosed with PAH (n = 247, 58 %) or CTEPH (n = 177, 42 %) between 1.1.2008 and 31.12.2019. Baseline characteristics of the patients are shown in Table 1. Mean age of the PAH patients was 57.5 (SD 16.3) years, and 181 (73 %) of the patients were female. Mean age of CTEPH patients was 63.3 (SD 13.5), and 91 (51 %) were female (Table 1). Patient characteristics for PAH subgroups (IPAH, HPAH, APAH [connective tissue disease], APAH [congenital heart disease]) and CTEPH subgroups (PEA-operated and non-operated) are shown in Supplementary Table 2.

**Table 1:** Characteristics of the study population.

	PAH	CTEPH
Baseline characteristics		
Patients, n	247	177
Age (year), mean (SD)	57.5 (16.3)	63.3 (13.5)
Sex (female), n (%)	181 (73)	91 (51)
Etiology of PAH, n (%)		
IPAH	102 (41)	
HPAH	9 (4)	
APAH, connective tissue disease	71 (29)	
APAH, congenital heart disease	24 (10)	
Other	41 (17)	
Comorbidities (at baseline*), n (%)		
Major cardiovascular comorbidities		
Treated hypertension (I10)	103 (42)	77 (44)
Diabetes (E10-E11)	53 (22)	26 (15)
Stroke (I63)	6 (2)	6 (3)
Ischaemic heart disease (I20-I25)	61 (25)	42 (24)
3 or more major cardiovascular comorbidities	47 (19)	15 (9)
Atrial fibrillation or flutter (I48)	47 (19)	21 (12)
Chronic kidney disease (N18)	13 (5)	<5
Lung disease		
Asthma (J45)	34 (14)	40 (23)
Parenchymal (J84)	25 (10)	0 (0)
COPD or emphysema (J44)	15 (6)	13 (7)
Rheumatic diseases		
Systemic sclerosis or CREST (M34)	42 (17)	0 (0)
Sjögren (M35.0)	18 (7)	0 (0)
Rheumatoid arthritis (M05-M06)	9 (4)	7 (4)
Other (including MCTD) (M35)	32 (13)	<5
Sleep apnoea (G47.3)	17 (7)	15 (9)
Cancer (C)	18 (7)	31 (18)
History of pulmonary embolism (I26, K93)	35 (14)	153 (86)
History of DVT (I80-I82)	6 (2)	29 (16)
PAH treatments received during follow-up**, n (%)		
Endothelin receptor antagonists	146 (59)	38 (21)
Phosphodiesterase 5 inhibitors	204 (83)	69 (39)
Guanylate cyclase stimulator	<5	62 (35)
Prostacyclin analogue	34 (14)	<5
Prostacyclin receptor agonist	15 (6)	0 (0)
Diuretics	205 (83)	135 (76)
Anticoagulation	182 (74)	177 (100)
Digoxin	51 (21)	18 (10)
CTEPH treatment, n (%)		
Pulmonary Endarterectomy		61 (35)
Balloon pulmonary angioplasty		41 (23)
Follow-up and survival		
Patients with full** follow-up, n (%)	199 (81)	135 (76)
1-year OS, alive, n (%)	227 (92)	166 (94)
5-year OS, alive, n (%)	112 (56)	97 (72)

CTEPH, Chronic Thromboembolic Pulmonary Hypertension; PAH, Pulmonary Arterial Hypertension. *Baseline period covers 5 years’ period pre-index date. **Follow-up covers 5 years’ periods pre- and 5 years post-index date.

### 3.2 Healthcare resource use

**Fig. 1:**
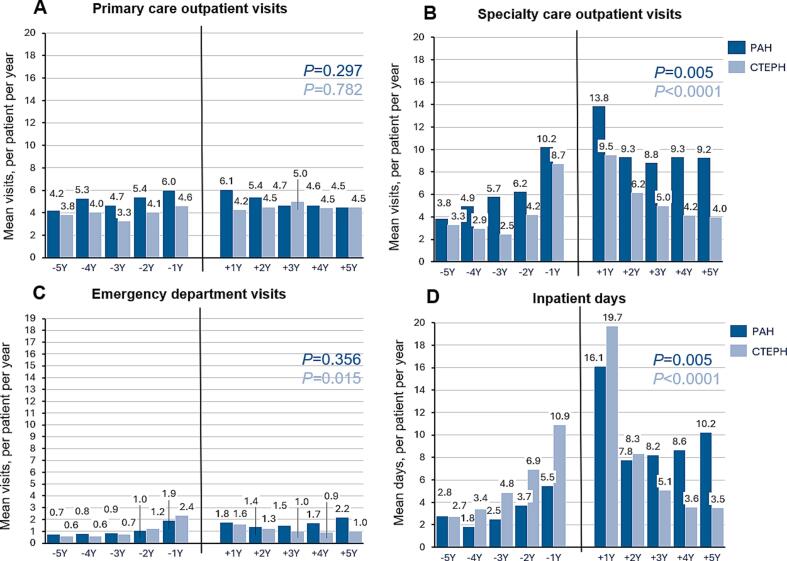
Healthcare resource use of the PAH and CTEPH patients 5 years before and 5 years after the diagnosis. Mean number of (A) primary outpatient care visits, (B) specialty care outpatient visits, (C) emergency department visits, and (D) days spent hospitalized per patient annually. The analysis includes only surviving patients with complete follow‐up data per year. Emergency department visits and inpatient days included both primary and specialty care visits and days, respectively. P-value denotes the significance of the longitudinal trend in analyzed HCRU over the five-year period following diagnosis (years + 1 to + 5). P-values with dark blue shown for PAH patients and with light blue for CTEPH patients.

Fig. 1 presents the mean annual healthcare resource use (HCRU) for PAH and CTEPH patients five years before and after diagnosis. PAH patients had higher annual specialty care visits than CTEPH patients both before and after diagnosis. This increase began four years prior diagnosis for PAH patients, compared to two years prior diagnosis for CTEPH patients, peaking at one-year post-diagnosis (13.8 visits for PAH, 9.5 for CTEPH). After this peak, specialty care visits declined, more markedly in CTEPH patients.

CTEPH patients had more annual inpatient days from four years before to two years post-diagnosis. Inpatient days for CTEPH decreased from 8.3 days at two years post-diagnosis to 3.5 days at five years post-diagnosis. For PAH patients, inpatient days increased slightly from 8.2 to 10.3 days in the same period. Inpatient days peaked one-year post-diagnosis (16.1 days for PAH, 19.7 for CTEPH).

CTEPH patients undergoing pulmonary endarterectomy (PEA) had higher inpatient days and specialty care visits for up to two years post-diagnosis compared to non-operated patients; these then decreased to levels lower than those in non-operated patients (Supplementary figure 2). PAH patients with connective tissue disease had more specialty care visits and inpatient days post-diagnosis compared to those with IPAH, HPAH, or congenital heart disease. Primary care visits were slightly higher for PAH patients than for CTEPH patients from five years before diagnosis until two years post-diagnosis, with no significant changes after diagnosis for either group (Fig. 1).

Three percent (N = 8) of PAH patients used homecare prior to diagnosis and 30 % (N = 75) used it after diagnosis; 4 % of CTEPH patients used homecare prior to diagnosis and 27 % (N = 47) after diagnosis. Homecare use increased from 3 % to 30 % in PAH patients and from 4 % to 27 % in CTEPH patients’ post-diagnosis. Only 3 % (N = 8) of PAH and 4 % (N = 7) of CTEPH patients used institutional care prior diagnosis, and 3 % of PAH (N = 7) and 5 % (N = 8) of CTEPH patients after diagnosis. Institutional care usage remained low for both groups before and after diagnosis. Detailed statistics are in Supplementary Table 3 and Supplementary Figure 2.

### 3.3 Drug utilization

**Fig. 2:**
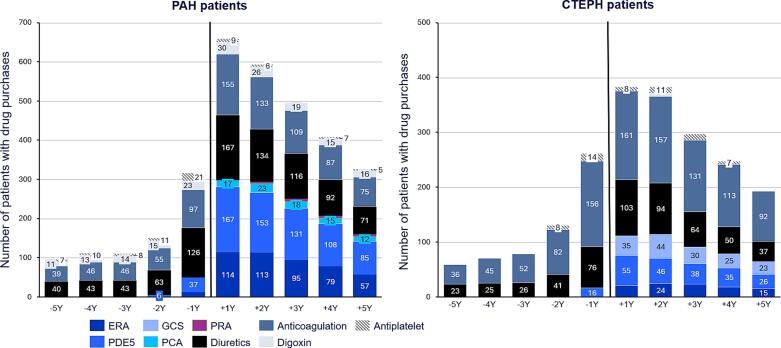
PAH-specific and supportive therapy drug use of the patients 5 years before and 5 years after the diagnosis for PAH and CTEPH patients. PAH-specific: ERA, Endothelin receptor antagonists; PDE5, Phosphodiesterase 5 inhibitors; GCS, Guanylate cyclase stimulator; PCA, Prostacyclin analogues; PRA, Prostacyclin receptor antagonist; supportive therapy: diuretics, anticoagulation, digoxin, antiplatelet.

Annual drug utilization of PAH and CTEPH patients is shown in Fig. 2. The most purchased PAH-specific drug for PAH was PDE5-inhibitor (PDE5i, N = 167 (74 %)), followed by endothelin receptor antagonists (ERA, N = 114 (50 %)); only a small proportion of patients purchased prostacyclin analogues (PCA, N = 17 (7 %) or prostacyclin receptor agonists (PRA, n=<5) 1 year after diagnosis. There were 19 (17 %) PAH patients with a positive vasoreactivity test who used CCBs for the treatment of PAH (data not shown). For CTEPH the most purchased drug was PDE5i (N = 55 (33 %)), followed by soluble guanylate cyclase stimulators (sGCs, N = 35 (21 %)) and ERAs (N = 21 (13 %)) one year after diagnosis.

Supportive therapy usage increased from two years before diagnosis. Diuretic use rose from 16 % to 51 % in PAH patients, and from 13 % to 43 % in CTEPH patients from five years to one year before diagnosis. Anticoagulation therapy usage increased from 16 % to 39 % in PAH patients and from 20 % to 88 % in CTEPH patients in the same period. Drug utilization peaked one year post-diagnosis and then decreased for both PAH-specific and supportive therapies. One year post-diagnosis, 97 % (N = 161) of CTEPH patients used anticoagulation medication. Supplementary Table 4 provides detailed annual drug utilization data for PAH and CTEPH subgroups.

### 3.4 Productivity loss

**Fig. 3:**
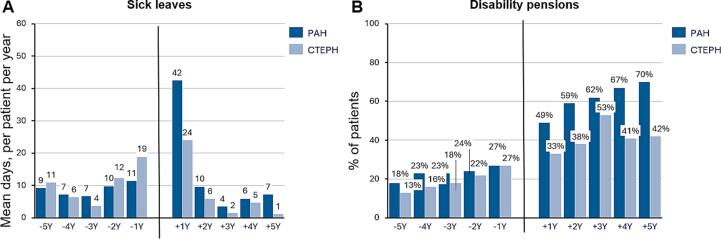
Annual sick leaves and disability pensions of the patients 5 years before and 5 years after the diagnosis. Mean annual number of (A) sick leave days per patient, and annual proportion of patients on disability pension (B).

The annual number of sick leave days and the proportion of patients on disability pension are shown in Fig. 3. During the study period, 42 % of PAH patients (n = 103) and 36 % of CTEPH patients (n = 63) took sick leave. Sick leave days began to increase two years before diagnosis. One year before diagnosis, CTEPH patients had more sick leave days (19 days) than PAH patients (11 days). The peak in sick leave days occurred one year after diagnosis, with PAH patients at 42 days and CTEPH patients at 24 days. Sick leave days declined after diagnosis, reaching a low three-year post-diagnosis.

At diagnosis, 58 % (n = 144) of PAH patients and 47 % (n = 83) of CTEPH patients were of working age. The proportion of working-age patients on disability pension rose from five years to one year before diagnosis (PAH: 18 % to 27 %; CTEPH: 13 % to 27 %). After diagnosis, the increase in disability pension was more significant for PAH patients (from 49 % to 70 %) compared to CTEPH patients (from 33 % to 42 %).

### 3.5 Total costs

**Fig. 4:**
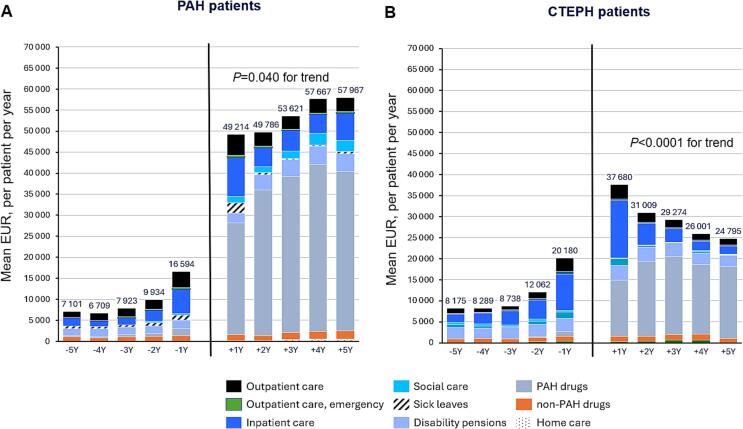
Total annual costs of the patients by category 5 years before and 5 years after the diagnosis. P-value denotes the significance of the longitudinal trend in total annual costs over the five-year period following diagnosis (years + 1 to + 5).

Mean annual costs by category for patients 5 years before and after diagnosis are shown in Fig. 4, with descriptive statistics in Supplementary Table 5. The mean total annual cost increased two years prior to diagnosis. From five years to one year before diagnosis, total costs rose by 133 % for PAH patients and 147 % for CTEPH patients. Outpatient and inpatient care were the highest cost drivers one to two years before diagnosis for both groups.

One-year post-diagnosis, total costs for PAH patients increased by 21 % (from 49,214 to 58,967 euros) until five years after diagnosis, while for CTEPH patients, costs decreased by 34 % (from 37,680 to 24,795 euros). Compared to other costs the drug costs were the highest post-diagnosis expenses for both PAH and CTEPH patients. Annual PAH-specific drug costs peaked at 4 years post-diagnosis, averaging 39,710 euros for PAH and 18,590 euros for CTEPH at 3 years post-diagnosis.

For PAH at 4 years after diagnosis, the highest mean costs/patient were for ERAs (18,238 EUR), followed by PCA (11,475 EUR), PDE5i (7,158 EUR), PRA (2,316 EUR), and sGCs (287 EUR). For CTEPH at 3 years after diagnosis, the highest costs were for sGCs (7,658 EUR), followed by ERAs (5,895 EUR), PDE5i (3,991 EUR), and PCA (102 EUR).

Inpatient care costs (included both costs of operative/invasive treatments and hospital stay) were higher for CTEPH patients (36 %) than for PAH patients (19 %) one year post-diagnosis. PEA-operated patients had higher inpatient care costs (57 %) compared to non-operated patients (22 %). Home care costs were minimal, accounting for 1 % for PAH and 2.6 % for CTEPH at their highest post-diagnosis. Mean annual costs by category for PAH and CTEPH subgroups 5 years before and after diagnosis are detailed in Supplementary Table 6.

The estimated total costs per patient for five years before and after diagnosis are shown in Table 2. PAH patients had 44 % higher costs after diagnosis compared to CTEPH patients (269,600€ for PAH vs. 151,600€ for CTEPH). This difference was mainly due to higher drug costs (181,100€ for PAH vs. 90,900€ for CTEPH), with outpatient and inpatient care costs also higher for PAH patients. Although the ESC/ERS 2015 guidelines promoted more aggressive treatment of PAH, total cost of patients diagnosed after 2015 were lower than those diagnosed before 2015, mainly due to lower drug cost (data not shown).

**Table 2:** The 5-year costs of the patients before and after index date.

	Before diagnosis, 5-year period	After diagnosis, 5-year period	Difference	P-value
PAH				
Total costs, Euros	48 200 (40 000–56 800)	269 600 (125 000–304 200)	221 400 (212 600–230 200)	<0.0001
Outpatient care	11 300 (8 000–14 700)	18 700 (16 200–21 100)	7 300 (4000–10700)	<0.0001
Emergency visits	1 200 (1 000–1 500)	1 700 (1 400–2 000)	400 (200–600)	0.0002
Inpatient care	13 100 (1 000–16 300)	34 100 (28 300–40 000)	20 900 (17 800–24 100)	<0.0001
Institutional care	800 (−600–2 200)	9 600 (−5 000–2 400)	8 800 (7 100–10 400)	<0.0001
Sick leaves	3 700 (2 800–4 600)	4 100 (3 000–5 300)	400 (−500–1 300)	0.386
Disability pensions	9 100 (6 400–11 900)	17 800 (13 100–22 400)	8 700 (5900–11400)	<0.0001
Drug utilization	8 900 (6 200–11 600)	181 100 (151 600–210 500)	172 200 (168 900–175 500)	<0.0001
Home care	0 (0–100)	2 000 (800–3 200)	1 900 (1 900–2 000)	<0.0001
CTEPH				
Total costs, Euros	57 300 (44 300–70 200)	151 600 (134 700–168 500)	94 300 (81 500–107 400)	<0.0001
Outpatient care	8 100 (6 500–9 700)	10 600 (9 400–11 900)	2 500 (900–4 100)	0.0002
Emergency visits	1 400 (1 200–1 600)	1 300 (1 000–1 500)	−100 (−300–100)	0.152
Inpatient care	20 900 (12 900–28 800)	28 400 (25 200–31 600)	7 500 (−400–15 500)	0.063
Institutional care	2 300 (−300–4 900)	600 (−400–1 500)	−1 700 (−43 00–800)	0.179
Sick leaves	3 800 (2 600–5 100)	2 800 (1 700–4 000)	−1 000 (−2 200–200)	0.109
Disability pensions	13 800 (8 200–19 400)	14 600 (6 500–22 800)	800 (−4 800–6 400)	0.771
Drug utilization	6 500 (4 900–8 100)	90 900 (76 900–105 000)	84 400 (82 500–86 300)	<0.0001
Home care	600 (−500–1 600)	2 100 (900–3 300)	1 600 (700–2 400)	0.001

Data are shown as mean (95% confidence interval), per patient, EUR. Zhao and Tian (ZT) estimator was used to adjust for censoring.

Before diagnosis, CTEPH patients had 16 % higher total costs than PAH patients, driven mainly by inpatient care (13,100€ for PAH vs. 20,900€ for CTEPH). Among PAH subtypes, patients with APAH related to connective tissue disease had the highest post-diagnosis costs (311,100€) compared to IPAH, HPAH (279,300€), and APAH related to congenital heart disease (239,400€) (Supplementary Table 7). Non-operated CTEPH patients had 26 % higher costs over five years after diagnosis compared to PEA-operated CTEPH patients (170,400€ vs. 125,700€), primarily due to higher drug costs (121,200€ for non-operated vs. 44,100€ for operated CTEPH) (Supplementary Table 7). The CTEPH patient who underwent BPA operation has slightly higher cost compared to non-operated (189,152€ vs. 164,102€). When comparing total cost and overall survival in the whole cohort, a higher total cost was associated with shorter overall survival, while a lower total cost was associated with longer overall survival (data not shown).

## 4 Discussion

To our knowledge, this is the most comprehensive study detailing costs for PAH and CTEPH, including primary care, specialty care, home care, institutional care, drug costs, and indirect costs. Our findings indicate that care costs for PAH were significantly higher than CTEPH during the five years post-diagnosis. For PAH patients, annual costs increased from the first to the fifth-year post-diagnosis, whereas for CTEPH patients, they decreased. In previous analysis of this cohort, we found that the median time from symptoms to diagnosis of PAH and CTEPH was 12 months [11]. Consistently, total annual cost of PAH significantly increased two years prior to diagnosis and were slightly higher for CTEPH patients.

Patients with PAH often require intensive management, contributing to both clinical burden and high mortality. Previous studies have evaluated PAH-related HCRU and costs post-diagnosis, identifying hospitalizations and drug expenses as primary cost drivers [7], [9], [16], consistent with our results. Studies has shown varying outcomes regarding drug and inpatient care costs. For instance, a chart review study across 11 European countries indicated medication expenses were the predominant cost component (84 %) [10], whereas a U.S. study based on insured claims data reported drug costs at only 10 % of total expenses [17]. Runheim et al. estimated the total societal cost for PAH care in Sweden over five years post-diagnosis was 176,600 euros, with medication costs comprising 63 % and inpatient care 16 % [9]. Our study estimated a similar cost distribution five years after diagnosis, with medication costs at 67 % and inpatient care at 13 % of the total 221,400 euros. Among PAH-specific and PAH-supportive medications, ERAs (18,238 EUR/year/patient) and PCA (11,475 EUR/year/patient) accounted for the highest costs four years after diagnosis. A difference in our study compared to Runheim's is the inclusion of primary care visits within the outpatient visits category, as well as homecare and institutional care. Our results showed annual primary care visits did not significantly change post-diagnosis, indicating patients are followed up in specialty care. Similarly [9], specialty care visits peaked one year after diagnosis and then decreased, as in Runheim's study.

To our knowledge, this study is the first to compare HCRU and costs across different PAH subgroups. We analyzed patients with IPAH/HPAH, APAH connective tissue diseases, and APAH congenital heart disease. Post-diagnosis, APAH connective tissue diseases incurred the highest costs due to higher drug and inpatient care expenses. Significant cost differences were in drug costs between APAH congenital heart disease and APAH connective tissue diseases, and inpatient days between IPAH/HPAH and APAH connective tissue diseases. Consistent with previous reports [17], [18] we found the prognosis of this PAH subgroup is poor with a 5-year survival of only 37 % compared to 62 % in IPAH/HPAH and 81 % in APAH congenital heart disease. This PAH subgroup is sicker and needs maximal medical treatment, hospitalization, and end-of-life care. The survival of PAH was 92 % and 56 % and of CTEPH 94 % and 72 % at 1 and 5 years, respectively. Shortened survival likely increases HCRU and costs for these patients.

In our study, we observed that the total cost of care for CTEPH over five years post-diagnosis was significantly lower compared to PAH—€151,600 for CTEPH versus €221,400 for PAH. The primary driver of this cost disparity was higher medication expenses in PAH patients. Indeed, there is ample trial evidence and guideline recommendations to support treatment of PAH with dual or triple therapies whereas in CTEPH PEA is the treatment of choice in operable patients and there is strong (class I) recommendation only for riociguat [19]. Additionally, the financial burden for CTEPH patients can vary depending on eligibility for and completion of pulmonary endarterectomy, which can significantly reduce the ongoing need for medication [10]. Among CTEPH patients, those who underwent PEA surgery (35 %) had lower overall costs (€125,700) compared to non-operated patients (€170,400), mainly due to reduced medication expenses. In contrast, UK studies reported similar costs between operated and non-operated patients [20], and Swedish studies showed higher costs for operated patients [8].Thus, this is to our knowledge the first paper to show that PEA −surgery, in addition to providing superior prognosis of CTEPH patients, is also economically beneficial. The discrepancy between our findings and those of Kjellström et al. [8] can be attributed to the greater difference in medication costs between operated and non-operated CTEPH patients in our study. In the Swedish study the mean hospitalization costs for PEA-patients was 4 times higher compared with non-PEA patients in the years 1 and 2 post −diagnosis, so the costs of PEA −surgery might at least partly explain the difference. Unlike PEA-operated CTEPH patients, those who underwent BPA had a slightly higher total cost compared to CTEPH patients who did not undergo the BPA procedure. In our study, in line with the treatment guidelines, riociguat was the most used PAH-specific drug in CTEPH and this might partially explain the higher drug costs. To our knowledge, this is the first real-world evidence describing the cost of riociguat in CTEPH. When comparing the total cost in the entire cohort, higher costs were associated with lower overall survival, indicating that patients in poorer condition require more expensive treatments.

The symptoms of PAH and CTEPH are often non-specific, causing diagnostic delays. HCRU for PAH patients increases significantly starting three years before diagnosis due to the time needed for diagnosis. Our study revealed that the total costs for PAH patients increased by 133 % from five years before diagnosis to one year prior, with noticeable increases already evident two years before diagnosis, driven mainly by outpatient and inpatient care. For IPAH/HPAH and APAH connective tissue disease, outpatient and inpatient services were the largest expenses before diagnosis, while for APAH congenital heart disease, drug costs and inpatient care were the primary expenses. Additionally, productivity loss costs were higher before diagnosis than after, consistent with Runheim et al. findings.

This may be explained by the high mortality rate among PAH patients and the fact that not all patients have follow-up data extending to five years post-diagnosis. Additionally, 70 % of working-age PAH patients were on disability pension five years post-diagnosis, representing significant long-term indirect costs and societal and individual challenges.

The strength of this study lies in its comprehensive analysis of societal costs associated with PAH and CTEPH, including their subtypes. This study includes a wide range of direct and indirect cost types including for example cost for primary care visits, homecare, and institutional care which have not been reported in the previous studies. The study had some limitations. It did not include a reference population to assess how age might affect cost development over the 5-year periods before and after diagnosis. Additionally, due to the low number of patients, it was not feasible to restrict the cohort to those who had full five years of follow-up data post-diagnosis. The results also present average values per patient for each resource and cost type. Since the proportion of patients utilizing each type of resource varies, individual costs can differ significantly, with some patients incurring much lower expenses than others.

## 5 Conclusion

In conclusion, the results indicate that PAH and CTEPH cause a substantial economic burden on patients and society. Significant differences are observed between PAH and CTEPH, as well as among PAH subtypes and between PEA-operated and non-operated CTEPH patients, particularly in drug costs.

## CRediT authorship contribution statement

**Markku Pentikäinen:** Writing – review & editing, Validation, Methodology, Investigation, Conceptualization. **Piia Simonen:** Writing – review & editing, Validation, Methodology, Investigation. **Pauliina Leskelä:** Writing – review & editing, Validation, Investigation. **Terttu Harju:** Writing – review & editing, Validation, Investigation. **Pertti Jääskeläinen:** Writing – review & editing, Validation, Investigation. **Christina Wennerström:** Writing – review & editing, Validation, Methodology, Investigation. **Nikolaj Bødker:** Writing – review & editing, Validation, Investigation. **Eija Heikkilä:** Writing – original draft, Validation, Project administration, Methodology, Conceptualization. **Mari Lahelma:** Writing – original draft, Validation, Methodology, Investigation, Formal analysis, Data curation. **Riikka-Leena Leskelä:** Writing – review & editing, Validation, Methodology, Investigation. **Airi Puhakka:** Writing – original draft, Validation, Methodology, Investigation. **Elina Heliövaara:** . **Katriina Kahlos:** . **Pentti Korhonen:** . **Tiina Kyllönen:** . **Kirsi Majamaa-Voltti:** . **Anu Turpeinen:** . **Helena Tuunanen:** . **Ville Vepsäläinen:** . **Tapani Vihinen:** .

## Funding

The study was supported by Janssen-Cilag Oy in Finland.

## Declaration of competing interest

The authors declare that they have no known competing financial interests or personal relationships that could have appeared to influence the work reported in this paper.

## Figures and Tables

**Figure:**
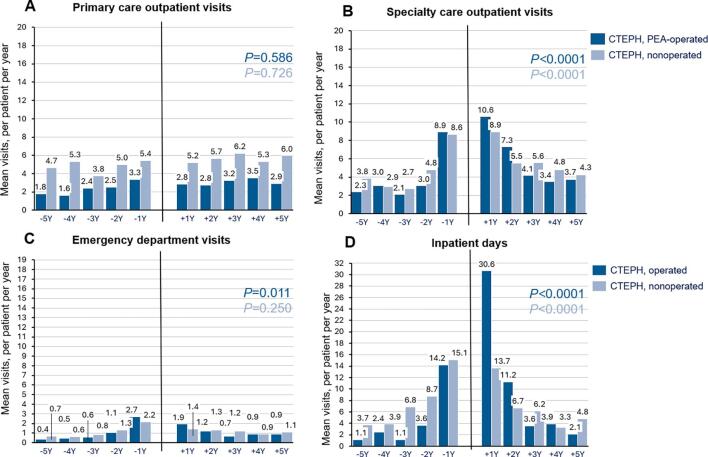


**Figure:**