# Supplementary material for: Economic burden of pulmonary arterial hypertension (PAH) and chronic thromboembolic pulmonary hypertension (CTEPH) in Finland
Source: Int J Cardiol Heart Vasc. 2024 Oct 22;55:101534. doi: 10.1016/j.ijcha.2024.101534 (PMC11539720; doi:10.1016/j.ijcha.2024.101534)
Supplement: Supplementary Data 1 [file mmc1.docx]

**Supplementary Table 1.** Data sources of the study.

| **Register and registry holder** | **Description** |
| --- | --- |
| Hilmo, Finnish Institute for Health and Welfare | Nationwide data on all inpatient episodes in public hospitals (primary and specialized care) as well as all outpatient visits (both scheduled appointments and emergency care visits) in specialized care, as well as deaths. Patients’ diagnoses recorded in ICD-10 codes, has nationwide coverage since 1998. |
| AvoHilmo, Finnish Institute for Health and Welfare | Nationwide data on outpatient visits in public primary care health centers with patients’ diagnoses recorded either by ICD-10 or ICPC-2 codes, has nationwide coverage since 2014. |
| SosiaaliHilmo, Finnish Institute for Health and Welfare | Nationwide data on social care episodes in home care, institutional care, and rehabilitation periods. SosiaaliHilmo has coverage since 1997. |
| Reimbursed medicine purchase information register, Kela | Information on prescription medicines reimbursed out of National Health Insurance, users of medication, doctors having prescribed medication, medication costs and reimbursements, generic substitution. Coverage since 1987. |
| Prescription Centre register, Kela | Information about medication (electronic prescriptions and dispensing’s at pharmacies). |
| Sick leave allowance payment register, Kela | Data on the number of reimbursed sick leave days and diagnosis related to them nationwide. The register covers all reimbursed sick leaves lasting continuously over 10 days. The register has national coverage since 1993. |
| Disability pensions register, Kela | Data on disability pensions covered by social insurance. Data includes start date of disability pension and monthly reimbursement. Coverage since 1997. |
| Pension register of work pensions, Finnish Centre for Pensions | The key figures for all new retirees and retired persons on an earnings-related pension in Finland including retirees on disability pension. |
| Digital and population information registry | Data on place of residence, date of birth and date of death. |

**Supplementary Table 2.** Characteristics of PAH and CTEPH subgroups.

|  | | **PAH subgroups** | | | | **CTEPH subgroups** | |
| --- | --- | --- | --- | --- | --- | --- | --- |
|  | **IPAH and HPAH** | **APAH, connective** | **APAH, congenital heart disease** | **CTEPH, PEA-operated** | | **CTEPH, non-operated** |  |
| **Baseline characteristics** | |  |  |  |  | |  |
| Patients, n | | 111 | 71 | 24 | 61 | | 116 |
| Age (year), mean (SD) | | 56.4 (17.0) | 63.5 (12.7) | 53.5 (19.2) | 57.2 (14.9) | | 66.6 (11.5) |
| Sex (female), n (%) | | 79 (71.2) | 55 (77.5) | 18 (75.0) | 30 (49.2) | | 61 (52.6) |
| Comorbidities (at baseline*), n (%) | |  |  |  |  | |  |
| Major cardiovascular comorbidities | |  |  |  |  | |  |
| Treated hypertension (I10) | | 50 (45.0) | 33 (46.5) | 5 (20.8) | 24 (39.3) | | 53 (45.7) |
| Diabetes (E10-E11) | | 28 (25.2) | 11 (15.5) | 5 (20.8) | 6 (9.8) | | 20 (17.2) |
| Stroke (I63) | | <5 | <5 | <5 | <5 | | <5 |
| Ischaemic heart disease (I20-I25) | | 32 (28.8) | 17 (23.9) | 6 (25.0) | 12 (19.7) | | 30 (25.9) |
| 3 or more major cardiovascular comorbidities | | 30 (27.0) | ≥5 | <5 | <5 | | ≥5 |
| Atrial fibrillation or flutter (I48) | | 19 (17.1) | 14 (19.7) | 8 (33.3) | <5 | | ≥5 |
| Chronic kidney disease (N18) | | 10 (9.0) | <5 | <5 | <5 | | <5 |
| Lung disease | |  |  |  |  | |  |
| Asthma (J45) | | 13 (11.7) | 8 (11.3) | 6 (25.0) | 12 (19.7) | | 28 (24.1) |
| Parenchymal (J84) | | <5 | 20 (28.2) | <5 | 0 (0.0) | | 0 (0.0) |
| COPD or emphysema (J44) | | 7 (6.3) | <5 | <5 | <5 | | >5 |
| Rheumatic diseases | |  |  |  |  | |  |
| Systemic sclerosis or CREST (M34) | | 0 (0.0) | 41 (57.7) | 0 (0.0) | 0 (0.0) | | 0 (0.0) |
| Sjögren (M35.0) | | <5 | 14 (19.7) | <5 | 0 (0.0) | | 0 (0.0) |
| Rheumatoid arthritis (M05-M06) | | <5 | 6 (8.5) | <5 | <5 | | ≥5 |
| Other (including MCTD) (M35) | | 5 (4.5) | 25 (35.2) | 0 (0.0) | <5 | | <5 |
| Sleep apnoea (G47.3) | | 10 (9.0) | 5 (7.0) | 0 (0.0) | <5 | | ≥5 |
| Valvular disease (moderate/severe) | |  |  |  |  | |  |
| Tricuspid regurgitation (I36) | | <5 | <5 | <5 | <5 | | ≥5 |
| Other (I34-I35, I37) | | ≥5 | 9 (12.7) | <5 | <5 | | ≥5 |
| Cancer (C) | | 9 (8.1) | 5 (7.0) | 0 (0.0) | 7 (11.5) | | 24 (20.7) |
| History of DVT (I80-I82) | | 0 (0.0) | <5 | <5 | 11 (18.0) | | 18 (15.5) |
| **PAH treatments received during follow-up**** n (%) | |  |  |  |  | |  |
| Endothelin receptor antagonists | | 71 (64.0) | 48 (67.6) | 10 (41.7) | 5 (8.2) | | 33 (28.5) |
| Phosphodiesterase 5 inhibitors | | 96 (86.5) | 56 (78.9) | 20 (83.3) | 16 (26.2) | | 53 (45.7) |
| Soluble Guanylate cyclase stimulator | | <5 | <5 | 0 (0.0) | 15 (24.6) | | 47 (40.5) |
| Prostacyclin analogue | | 18 (16.2) | ≥5 | <5 | <5 | | <5 |
| Prostacyclin receptor agonist | | ≥5 | 8 (11.3) | <5 | 0 (0.0) | | 0 (0.0) |
| Diuretics | | 85 (76.6) | 62 (87.3) | 18 (75.0) | 43 (70.5) | | 92 (79.3) |
| Anticoagulation | | 86 (77.5) | 48 (67.6) | 21 (87.5) | 61 (100.0) | | 116 (100.0) |
| Digoxin | | 24 (21.6) | 11 (15.5) | 8 (33.3) | 7 (11.5) | | 11 (9.5) |
| **CTEPH treatment,** n (%) | |  |  |  |  | |  |
| Pulmonary Endarterectomy | | – | – | – | 61 (35) | | 0 (0.0) |
| Balloon pulmonary angioplasty | | – | – | – | 9 (14.8) | | 32 (27.6) |
| **Follow-up and survival** | |  |  |  |  | |  |
| Patients with full** follow-up, n (%) | | 86 (77.5) | 57 (80.3) | 21 (87.5) | 51 (83.6) | | 84 (72.4) |
| 1-year OS, alive, n (%) | | 103 (92.8) | 62 (87.3) | 24 (100.0) | 59 (96.7) | | 107 (92.2) |
| 5-year OS, alive, n (%) | | 53 (61.6) | 21 (36.8) | 17 (81.0) | 46 (90.2) | | 51 (60.7) |

APAH, Associated pulmonary arterial hypertension; CTEPH, Chronic Thromboembolic Pulmonary Hypertension; HPAH, Heritable Pulmonary Arterial Hypertension; IPAH, Idiopathic Pulmonary Arterial Hypertension; OS, overall survival. *Baseline period covers 5 years’ period pre-index date. **Follow-up covers 5 years’ periods pre- and 5 years post-index date.

**Supplementary Table 3.** Annual HCRU and other elements affecting societal costs 5 years before and 5 years after the diagnosis the PAH and CTEPH patients.

|  | **Before diagnosis** (years) | | | | | **After diagnosis** (years) | | | | |
| --- | --- | --- | --- | --- | --- | --- | --- | --- | --- | --- |
| **PAH** | -5 | -4 | -3 | -2 | -1 | +1 | +2 | +3 | +4 | +5 |
| HCRU, mean (SD) visits/days |  |  |  |  |  |  |  |  |  |  |
| Primary care outpatient visits | 4.19 (17.13) | 5.26 (27.09) | 4.68 (21.17) | 5.38 (25.95) | 6.03 (24.22) | 6.06 (26.45) | 5.41 (11.91) | 4.68 (6.52) | 4.64 (6.94) | 4.52 (6.4) |
| Secondary care outpatient visits | 3.81 (7.23) | 4.92 (13.88) | 5.74 (17.51) | 6.21 (18.36) | 10.17 (24.92) | 13.83 (22.03) | 9.26 (10.55) | 8.81 (11.71) | 9.28 (14.14) | 9.21 (12.7) |
| Emergency department visits | 0.75 (1.75) | 0.79 (2.03) | 0.87 (2.04) | 1.04 (2.21) | 1.92 (2.96) | 1.76 (3.04) | 1.37 (2.69) | 1.5 (3.23) | 1.7 (3.83) | 2.2 (6.67) |
| Inpatient days | 2.89 (11.1) | 1.95 (6.57) | 2.57 (15.42) | 3.89 (13.35) | 9.68 (13.14) | 16.13 (19.99) | 7.78 (15.49) | 8.2 (20.98) | 8.64 (22.05) | 10.23 (26.12) |
| Institutional care days | 0.26 (2.98) | 0.26 (4.01) | 0 (0) | 1.02 (15.97) | 0.92 (11.04) | 3.75 (40.15) | 3.66 (36.03) | 4.71 (41.59) | 7.05 (61.35) | 6.53 (48.62) |
| Home care days | 0.37 (2.17) | 0.03 (0.21) | 0.03 (0.27) | 0.25 (1.91) | 0.6 (3.61) | 4.53 (20.18) | 4.3 (21.93) | 10.65 (56.39) | 15.57 (87.96) | 14.71 (69.58) |
| Sick leave days | 9.28 (36.16) | 8.13 (33.99) | 7.17 (30.2) | 10.85 (41.12) | 17.67 (43.65) | 42.46 (81.37) | 9.66 (33.43) | 3.56 (20.59) | 5.98 (30.14) | 7.22 (31.82) |
| **CTEPH** |  |  |  |  |  |  |  |  |  |  |
| HCRU, mean (SD) visits/days |  |  |  |  |  |  |  |  |  |  |
| Primary care outpatient visits | 3.82 (7.63) | 4.02 (7.53) | 3.26 (5.24) | 4.08 (6.12) | 4.61 (6.03) | 4.25 (8.48) | 4.52 (7.49) | 5.02 (8.11) | 4.48 (8.18) | 4.52 (6.6) |
| Secondary care outpatient visits | 3.31 (8.76) | 2.94 (6.74) | 2.47 (5.48) | 4.18 (6.69) | 8.71 (13.8) | 9.5 (9.38) | 6.16 (11.3) | 4.97 (5.67) | 4.16 (5.85) | 3.98 (6) |
| Emergency department visits | 0.56 (1.4) | 0.59 (1.35) | 0.74 (1.89) | 1.23 (2.03) | 2.36 (2.96) | 1.61 (3.19) | 1.25 (2.97) | 1.01 (2.12) | 0.88 (1.82) | 1 (1.68) |
| Inpatient days | 2.77 (9.4) | 3.38 (10.89) | 4.83 (29.44) | 6.98 (24.27) | 14.8 (18.08) | 19.69 (23.22) | 8.32 (17.36) | 5.09 (10.89) | 3.56 (10.78) | 3.52 (10.35) |
| Institutional care days | 5.67 (45.89) | 5.88 (42.27) | 3.94 (34.09) | 4.42 (38.72) | 3.34 (30.24) | 0.34 (2.61) | 0.31 (3.41) | 1.05 (11.75) | 4.56 (49.93) | 3.77 (37.16) |
| Home care days | 0.19 (1.52) | 0.12 (0.99) | 0.09 (0.57) | 6.42 (70.11) | 8.79 (91.07) | 6.73 (52.88) | 9.49 (81.29) | 17.47 (103.2) | 18.16 (122.6) | 1.53 (8.01) |
| Sick leave days | 10.89 (44.6) | 6.36 (26.74) | 3.73 (19.76) | 12.23 (44.33) | 21.18 (48.42) | 24.01 (59.43) | 5.83 (27.24) | 1.59 (10.53) | 4.73 (28.06) | 1.23 (7.1) |

Data are as mean (SD) number of visits (or days for inpatient, social or sick leave days) per patient per year. CTEPH, Chronic Thromboembolic Pulmonary Hypertension; PAH, Pulmonary Arterial Hypertension.

**Supplementary Table 4.** Annual HCRU and other elements affecting societal costs 5 years before and 5 years after the diagnosis of the PAH and CTEPH subgroups.

|  | **Before diagnosis** | | | | | | **After diagnosis** | | | | |  |
| --- | --- | --- | --- | --- | --- | --- | --- | --- | --- | --- | --- | --- |
| **IPAH and HPAH** | -5 | -4 | -3 | -2 | -1 | +1 | | +2 | +3 | +4 | +5 | *P*-value^1^ |
| Primary care outpatient visits | 1.37 (1.61) | 2.46 (4.86) | 1.78 (2.9) | 2.63 (3.53) | 4.08 (4.83) | 3.28 (4.71) | | 3.49 (4.25) | 4.23 (5.67) | 4.48 (6.34) | 4.64 (5.54) | 0.065 |
| Secondary care outpatient visits | 2.86 (6.8) | 5.25 (19.04) | 5.86 (23.78) | 6.38 (26.22) | 11.05 (36.28) | 13.15 (30.41) | | 7.43 (7.9) | 7.54 (8.44) | 6.62 (6.96) | 7.91 (8.56) | **0.033** |
| Emergency department visits | 0.6 (1.55) | 0.73 (1.78) | 0.59 (1.73) | 0.93 (2.32) | 1.77 (3.3) | 1.5 (3.16) | | 1.05 (1.97) | 1.32 (2.27) | 1.25 (2.27) | 1.98 (3.68) | 0.397 |
| Inpatient days | 3.32 (13.16) | 1.65 (4.74) | 1.25 (3.22) | 3.44 (10.16) | 10.04 (14.56) | 15.43 (22.68) | | 5.19 (10.54) | 3.48 (7.46) | 3.38 (9.37) | 7.13 (22.43) | **<0.001** |
| **APAH, connective** |  |  |  |  |  |  | |  |  |  |  |  |
| Primary care outpatient visits | 2.22 (3.77) | 2.49 (4.94) | 2.66 (4.48) | 3.06 (4.96) | 4.8 (7.89) | 4.98 (8.92) | | 6.78 (11.49) | 4.54 (5.4) | 4.16 (4.91) | 5.76 (10.26) | 0.728 |
| Secondary care outpatient visits | 5.75 (7.94) | 6.13 (7.9) | 7.01 (8.63) | 8.04 (7.89) | 10.45 (7.04) | 17.21 (10.74) | | 12.58 (9.94) | 10.77 (9.07) | 11.91 (10.42) | 10.86 (10.33) | **0.003** |
| Emergency department visits | 1.11 (2.28) | 0.73 (1.93) | 0.96 (2.13) | 1.03 (2.13) | 1.9 (2.7) | 2.48 (3.23) | | 2.07 (3.89) | 1.44 (2.06) | 1.72 (2.77) | 1.24 (1.37) | 0.056 |
| Inpatient days | 1.9 (4.65) | 2.21 (7.56) | 5.39 (28.1) | 4.68 (18.1) | 8.83 (11.29) | 15.1 (14.08) | | 11.4 (19.08) | 16.21 (36.68) | 10.03 (13.46) | 9.29 (14.63) | 0.297 |
| **APAH, congenital heart disease** |  |  |  |  |  |  | |  |  |  |  |  |
| Primary care outpatient visits | 1.17 (1.83) | 1.22 (2.59) | 1.17 (1.59) | 3.08 (3.87) | 3.73 (2.91) | 4.4 (5) | | 3.73 (4.92) | 5.69 (5.11) | 6.11 (8.02) | 4.88 (5) | 0.470 |
| Secondary care outpatient visits | 4.58 (9.19) | 1.83 (5.04) | 3.33 (5.1) | 4.04 (7.06) | 7 (7.66) | 9 (11.33) | | 5.9 (6.28) | 6.48 (6.1) | 7.67 (9.89) | 6.88 (8.87) | 0.622 |
| Emergency department visits | 0.42 (0.88) | 0.29 (0.69) | 1 (1.74) | 1.25 (2.01) | 1.62 (2.76) | 0.5 (1.18) | | 1.14 (1.68) | 3.33 (7.4) | 4.44 (8.78) | 5.41 (15.59) | **0.023** |
| Inpatient days | 1.88 (5.14) | 1.08 (2.65) | 2.21 (5.68) | 3.29 (5.08) | 7.04 (11.12) | 14 (11.46) | | 6.67 (15.55) | 9.9 (19.99) | 16.17 (33.81) | 11.12 (27.99) | 0.867 |
| **CTEPH, PEA-operated** |  |  |  |  |  |  | |  |  |  |  |  |
| Primary care outpatient visits | 1.76 (4.9) | 1.62 (2.86) | 2.38 (4.4) | 2.53 (4.41) | 3.34 (4.17) | 2.84 (3.75) | | 2.76 (3.55) | 3.22 (4.33) | 3.5 (5.67) | 2.91 (3.7) | 0.586 |
| Secondary care outpatient visits | 2.34 (6.91) | 2.97 (7.78) | 2.05 (5.99) | 3 (6.07) | 8.85 (11.39) | 10.58 (10.68) | | 7.27 (16.09) | 4.12 (4.3) | 3.43 (5.51) | 3.67 (7.16) | **<0.001** |
| Emergency department visits | 0.36 (0.75) | 0.49 (1.07) | 0.56 (1.09) | 1.07 (1.77) | 2.72 (2.93) | 1.93 (3.18) | | 1.19 (2.63) | 0.7 (1.23) | 0.89 (1.78) | 0.89 (1.74) | **0.011** |
| Inpatient days | 1.07 (3.52) | 2.43 (9.12) | 1.07 (2.63) | 3.64 (9.15) | 14.21 (15.15) | 30.64 (21.52) | | 11.22 (21.55) | 3.55 (6.89) | 3.88 (8.82) | 2.09 (6.32) | **<0.001** |
| **CTEPH, non-operated** |  |  |  |  |  |  | |  |  |  |  |  |
| Primary care outpatient visits | 4.65 (8.39) | 5.31 (8.86) | 3.77 (5.63) | 5 (6.81) | 5.43 (6.87) | 5.21 (10.48) | | 5.66 (9.01) | 6.23 (9.71) | 5.34 (9.83) | 5.96 (8.18) | 0.726 |
| Secondary care outpatient visits | 3.82 (9.57) | 2.93 (6.15) | 2.7 (5.21) | 4.79 (6.94) | 8.63 (14.96) | 8.91 (8.57) | | 5.53 (7.34) | 5.55 (6.4) | 4.8 (6.1) | 4.25 (4.77) | **<0.001** |
| Emergency department visits | 0.67 (1.63) | 0.64 (1.48) | 0.84 (2.19) | 1.32 (2.16) | 2.17 (2.96) | 1.43 (3.2) | | 1.29 (3.16) | 1.23 (2.54) | 0.88 (1.86) | 1.1 (1.64) | 0.250 |
| Inpatient days | 3.67 (11.25) | 3.89 (11.72) | 6.81 (36.21) | 8.73 (29.14) | 15.1 (19.51) | 13.65 (21.97) | | 6.67 (14.31) | 6.15 (12.87) | 3.28 (12.3) | 4.8 (12.88) | **<0.001** |

Data are as mean (SD). ^1^*P*-value denotes the significance of the trend in HCRU parameter over the five-year period following diagnosis (years +1 to +5). (years +1 to +5). APAH, Associated pulmonary arterial hypertension; CTEPH, Chronic Thromboembolic Pulmonary Hypertension; HCRU, healthcare resource utilization; HPAH, Heritable Pulmonary Arterial Hypertension; IPAH, Idiopathic Pulmonary Arterial Hypertension; PAH, Pulmonary Arterial Hypertension.

**Supplementary Table 5.** Annual costs 5 by category 5 years before and 5 years after the diagnosis the PAH and CTEPH patients. Data are as mean (SD) 1000 EUR per year.

|  | **Before diagnosis** (years) | | | | | **After diagnosis** (years) | | | | |
| --- | --- | --- | --- | --- | --- | --- | --- | --- | --- | --- |
| **PAH** | -5 | -4 | -3 | -2 | -1 | +1 | +2 | +3 | +4 | +5 |
| Outpatient visits | 1.37 (2.49) | 1.78 (4.97) | 2.1 (6.35) | 2.31 (6.7) | 3.78 (9.1) | 5.03 (7.99) | 3.42 (3.65) | 3.23 (3.97) | 3.42 (4.84) | 3.37 (4.29) |
| Emergency department visits | 0.17 (0.36) | 0.17 (0.45) | 0.19 (0.48) | 0.23 (0.46) | 0.49 (0.72) | 0.46 (0.89) | 0.28 (0.55) | 0.28 (0.46) | 0.27 (0.51) | 0.33 (0.8) |
| Inpatient care | 1.88 (7.03) | 1.27 (4.11) | 1.69 (10.41) | 2.57 (9.38) | 5.7 (7.72) | 9.29 (12.09) | 4.49 (9.39) | 4.84 (12.01) | 4.47 (9.37) | 6.43 (19.64) |
| Institutional care | 0.03 (0.29) | 0.02 (0.39) | 0 (0) | 0.41 (6.48) | 0.34 (4.47) | 1.52 (16.29) | 1.46 (14.61) | 1.85 (16.85) | 2.86 (24.9) | 2.65 (19.73) |
| Home care | 0.01 (0.08) | 0 (0.01) | 0 (0.01) | 0.01 (0.07) | 0.02 (0.14) | 0.17 (0.76) | 0.16 (0.82) | 0.4 (2.11) | 0.58 (3.3) | 0.55 (2.61) |
| Sick leaves | 0.65 (2.48) | 0.51 (2.09) | 0.52 (2.01) | 0.76 (2.61) | 1.26 (3.2) | 2.37 (4.63) | 0.57 (2) | 0.24 (1.24) | 0.26 (1.31) | 0.61 (2.65) |
| Disability pension | 1.63 (4.53) | 1.86 (4.74) | 1.83 (4.82) | 1.83 (4.86) | 1.96 (4.93) | 2.43 (5.41) | 3.58 (6.89) | 3.97 (7.14) | 4.25 (7.4) | 4.13 (7.5) |
| Drugs, PAH-related | 0.32 (3.18) | 0.2 (1.36) | 0.53 (4.12) | 0.65 (5.27) | 1.75 (6.82) | 26.52 (26.52) | 34.65 (37.2) | 37.17 (42.03) | 39.71 (53.78) | 37.96 (56.36) |
| Drugs, non-PAH-related | 1.04 (3.33) | 0.88 (2.26) | 1.06 (2.85) | 1.16 (2.99) | 1.29 (3.42) | 1.43 (4.49) | 1.18 (3.09) | 1.66 (5.55) | 1.83 (4.72) | 1.92 (5.47) |
| **CTEPH** |  |  |  |  |  |  |  |  |  |  |
| Outpatient visits | 1.23 (3.15) | 1.12 (2.41) | 0.95 (1.89) | 1.58 (2.39) | 3.24 (5) | 3.46 (3.31) | 2.31 (3.94) | 1.95 (2) | 1.64 (2.12) | 1.6 (2.2) |
| Emergency department visits | 0.14 (0.32) | 0.13 (0.29) | 0.18 (0.38) | 0.32 (0.49) | 0.62 (0.69) | 0.39 (0.69) | 0.26 (0.58) | 0.23 (0.61) | 0.17 (0.37) | 0.19 (0.39) |
| Inpatient care | 1.91 (6.82) | 2.49 (8.57) | 3.31 (23.39) | 4.51 (18.58) | 8.64 (11.02) | 13.7 (16.7) | 5.23 (12.09) | 3.17 (6.71) | 2.24 (5.86) | 1.91 (5.38) |
| Institutional care | 0.54 (4.34) | 0.58 (4.13) | 0.39 (3.31) | 0.44 (3.9) | 0.36 (3.29) | 0.04 (0.33) | 0.03 (0.33) | 0.06 (0.61) | 0.23 (2.57) | 0.19 (1.91) |
| Home care | 0.01 (0.06) | 0 (0.04) | 0 (0.02) | 0.24 (2.63) | 0.33 (3.41) | 0.25 (1.98) | 0.36 (3.05) | 0.65 (3.87) | 0.68 (4.59) | 0.06 (0.3) |
| Sick leaves | 0.73 (3.13) | 0.46 (1.9) | 0.33 (1.67) | 0.83 (2.96) | 1.49 (3.3) | 1.75 (4.42) | 0.42 (1.75) | 0.13 (0.7) | 0.34 (2.18) | 0.12 (0.66) |
| Disability pension | 2.69 (7.43) | 2.45 (6.59) | 2.62 (7.07) | 2.95 (11.15) | 3.11 (11.29) | 3.37 (11.53) | 3.36 (11.62) | 3.19 (12.35) | 2.76 (8.53) | 2.62 (6.91) |
| Drugs, PAH-related | 0.03 (0.08) | 0.07 (0.46) | 0.05 (0.13) | 0.18 (0.55) | 1.25 (2.9) | 13.47 (16.43) | 17.92 (19.06) | 18.59 (20.99) | 16.51 (19.73) | 17.08 (22.65) |
| Drugs, non-PAH-related | 0.9 (2.02) | 0.98 (2.27) | 0.9 (1.96) | 1 (2.12) | 1.15 (2.32) | 1.24 (2.78) | 1.13 (2.03) | 1.3 (2.57) | 1.43 (5.06) | 1.03 (2.11) |

Data are as mean (SD) number of visits (or days for inpatient, social or sick leave days) per patient per year. CTEPH, Chronic Thromboembolic Pulmonary Hypertension; PAH, Pulmonary Arterial Hypertension.

**Supplementary Table 6.** Annual costs 5 by category 5 years before and 5 years after the diagnosis the PAH and CTEPH patients. Data are as mean (SD) 1000 EUR per year.

|  | **Before diagnosis** (years) | | | | | **After diagnosis** (years) | | | | |
| --- | --- | --- | --- | --- | --- | --- | --- | --- | --- | --- |
| **IPAH and HPAH** | -5 | -4 | -3 | -2 | -1 | +1 | +2 | +3 | +4 | +5 |
| Outpatient visits | 1 (2.3) | 1.84 (6.81) | 2.13 (8.63) | 2.34 (9.57) | 4.09 (13.22) | 4.75 (11.08) | 2.72 (2.76) | 2.8 (2.9) | 2.54 (2.47) | 2.96 (2.97) |
| Emergency department visits | 0.16 (0.39) | 0.15 (0.43) | 0.13 (0.36) | 0.21 (0.55) | 0.49 (0.85) | 0.42 (1.02) | 0.21 (0.42) | 0.25 (0.38) | 0.19 (0.34) | 0.28 (0.39) |
| Inpatient care | 2.16 (8.22) | 1.19 (3.59) | 0.86 (2.34) | 2.34 (7.3) | 5.5 (7.44) | 8.63 (13.84) | 2.77 (5.55) | 2.08 (4.59) | 2.12 (5.81) | 3.56 (8.82) |
| Institutional care | 0.02 (0.24) | 0 (0) | 0 (0) | 0.92 (9.67) | 0.62 (6.55) | 1.91 (19.35) | 1.6 (15.4) | 2.08 (18.45) | 4.03 (32.26) | 2.79 (20.35) |
| Home care | 0.03 (0.12) | 0 (0.01) | 0 (0.01) | 0.02 (0.1) | 0.01 (0.03) | 0.22 (0.97) | 0.11 (0.48) | 0.22 (1.09) | 0.18 (1.31) | 0.58 (2.54) |
| Sick leaves | 0.54 (1.82) | 0.44 (1.54) | 0.45 (1.51) | 0.73 (2.53) | 1.29 (3.41) | 2.74 (4.84) | 0.57 (2.16) | 0.36 (1.55) | 0.33 (1.23) | 0.65 (2.85) |
| Disability pension | 1.3 (4.29) | 1.28 (3.92) | 1.18 (3.78) | 1.24 (3.85) | 1.51 (4.27) | 1.95 (5.11) | 3.34 (6.16) | 3.73 (6.58) | 3.97 (6.8) | 3.27 (6.28) |
| Drugs, PAH-related | 0.08 (0.57) | 0.09 (0.74) | 0.38 (3.08) | 0.94 (7.3) | 2.09 (8.29) | 31.8 (31.46) | 41.67 (46.14) | 41.98 (46.02) | 44.18 (52.15) | 42.75 (54.54) |
| Drugs, non-PAH-related | 1.29 (4.43) | 1.11 (3.11) | 1.42 (3.97) | 1.33 (3.95) | 1.5 (4.67) | 1.52 (5.94) | 1.08 (3.99) | 1.34 (4.36) | 1.75 (4.41) | 1.43 (3.82) |
| **APAH, connective** |  |  |  |  |  |  |  |  |  |  |
| Outpatient visits | 2.02 (2.66) | 2.14 (2.58) | 2.44 (2.58) | 2.88 (2.71) | 3.78 (2.38) | 6.15 (3.6) | 4.7 (3.45) | 3.95 (3.13) | 4.27 (3.52) | 3.98 (3.54) |
| Emergency department visits | 0.23 (0.4) | 0.14 (0.36) | 0.17 (0.36) | 0.24 (0.4) | 0.43 (0.51) | 0.58 (0.8) | 0.39 (0.63) | 0.33 (0.53) | 0.33 (0.5) | 0.24 (0.38) |
| Inpatient care | 1.28 (3.3) | 1.49 (5) | 3.52 (18.99) | 3.08 (13.31) | 5.54 (7.77) | 8.58 (7.57) | 6.87 (11.76) | 9.58 (20.32) | 5.49 (6.86) | 4.68 (7.2) |
| Institutional care | 0 (0) | 0 (0) | 0 (0) | 0 (0) | 0 (0) | 0 (0) | 0.01 (0.05) | 0 (0) | 0 (0) | 0 (0) |
| Home care | 0.01 (0.03) | 0 (0) | 0 (0.01) | 0.01 (0.06) | 0.03 (0.15) | 0.18 (0.65) | 0.21 (0.72) | 0.4 (1.53) | 0.62 (2.56) | 0.29 (1.04) |
| Sick leaves | 0.61 (2.12) | 0.85 (2.99) | 0.84 (3.04) | 0.4 (2.01) | 0.8 (2.57) | 1.13 (3.14) | 0.61 (2.17) | 0.2 (1.23) | 0.37 (2.08) | 1.2 (4.01) |
| Disability pension | 2.16 (5.08) | 2.56 (5.33) | 2.59 (5.76) | 2.71 (5.96) | 2.6 (5.75) | 3.1 (6.01) | 4.21 (8.97) | 4.52 (9.37) | 5.22 (9.78) | 5.16 (10.98) |
| Drugs, PAH-related | 0.34 (2.33) | 0.35 (2.05) | 0.38 (2.27) | 0.32 (2.08) | 1.12 (3.36) | 26.54 (18.84) | 34.99 (27.59) | 45.83 (44.78) | 48.95 (63.58) | 51.14 (71.9) |
| Drugs, non-PAH-related | 0.98 (2.49) | 0.75 (1.1) | 0.82 (1.23) | 1.21 (2.28) | 1.32 (2.22) | 1.76 (3.48) | 1.53 (2.46) | 3.06 (9.45) | 3.04 (7.3) | 4 (10.49) |
| **APAH, congenital heart disease** |  |  |  |  |  |  |  |  |  |  |
| Outpatient visits | 1.52 (3.17) | 0.67 (1.87) | 1.16 (1.8) | 1.46 (2.44) | 2.6 (2.76) | 3.32 (4.15) | 2.12 (2.19) | 2.37 (2.05) | 2.92 (3.3) | 2.6 (3.04) |
| Emergency department visits | 0.14 (0.27) | 0.08 (0.2) | 0.27 (0.53) | 0.28 (0.42) | 0.38 (0.58) | 0.16 (0.4) | 0.3 (0.51) | 0.44 (0.77) | 0.59 (1.01) | 0.68 (1.79) |
| Inpatient care | 1.23 (3.12) | 0.67 (1.49) | 1.53 (3.78) | 2.35 (4.1) | 4.32 (7.02) | 9.03 (6.96) | 3.2 (6.7) | 6.26 (13.19) | 7.82 (15.05) | 5.32 (12.24) |
| Institutional care | 0 (0) | 0 (0) | 0 (0) | 0 (0) | 0.56 (2.73) | 6.17 (30.24) | 7.05 (32.32) | 7.05 (32.32) | 8.23 (34.91) | 8.74 (36.02) |
| Home care | 0 (0) | 0 (0) | 0 (0) | 0 (0) | 0.1 (0.37) | 0.17 (0.64) | 0.58 (2.24) | 1.34 (5.25) | 1.16 (4.69) | 1.18 (4.86) |
| Sick leaves | 1.44 (5.23) | 0.1 (0.49) | 0.34 (1.27) | 0.99 (2.67) | 1.29 (2.84) | 2.91 (5.62) | 0.28 (1.13) | 0.23 (0.74) | 0.26 (0.82) | 0.27 (0.76) |
| Disability pension | 1.52 (3.17) | 0.67 (1.87) | 1.16 (1.8) | 1.46 (2.44) | 2.6 (2.76) | 3.32 (4.15) | 2.12 (2.19) | 2.37 (2.05) | 2.92 (3.3) | 2.6 (3.04) |
| Drugs, PAH-related | 1.94 (9.31) | 0.44 (1.99) | 2.23 (10.76) | 1.11 (5.17) | 2.91 (10.09) | 14.6 (18.18) | 23.23 (21.64) | 26.02 (34.76) | 33.83 (37.49) | 30.2 (60.57) |
| Drugs, non-PAH-related | 0.49 (0.67) | 0.55 (0.91) | 0.48 (0.73) | 0.59 (0.89) | 0.74 (1.22) | 0.7 (1.26) | 0.53 (0.65) | 0.53 (0.68) | 0.63 (0.68) | 0.48 (0.46) |
|  |  |  |  |  |  |  |  |  |  |  |
|  | **Before diagnosis** (years) | | | | | **After diagnosis (years)** | | | | |
| **CTEPH, PEA-operated** | -5 | -4 | -3 | -2 | -1 | +1 | +2 | +3 | +4 | +5 |
| Outpatient visits | 0.81 (2.31) | 1.08 (2.71) | 0.79 (2) | 1.12 (2.03) | 3.26 (4.07) | 3.79 (3.76) | 2.6 (5.53) | 1.58 (1.57) | 1.33 (1.8) | 1.44 (2.65) |
| Emergency department visits | 0.1 (0.25) | 0.13 (0.29) | 0.13 (0.25) | 0.3 (0.55) | 0.67 (0.7) | 0.49 (0.82) | 0.26 (0.53) | 0.13 (0.25) | 0.18 (0.37) | 0.17 (0.36) |
| Inpatient care | 0.83 (2.83) | 1.84 (7.39) | 0.64 (1.53) | 2.37 (6.7) | 8.74 (9.75) | 24.79 (18.21) | 7.81 (16.62) | 2.49 (5.76) | 3.09 (7.19) | 1.37 (4.11) |
| Institutional care | 0 (0) | 0.04 (0.31) | 0.11 (0.87) | 0.09 (0.69) | 0 (0) | 0 (0) | 0.07 (0.55) | 0 (0) | 0 (0) | 0 (0) |
| Home care | 0 (0.01) | 0 (0) | 0 (0) | 0 (0) | 0 (0.01) | 0.07 (0.34) | 0.05 (0.21) | 0.04 (0.21) | 0.06 (0.29) | 0.04 (0.24) |
| Sick leaves | 1.21 (4.57) | 0.46 (1.35) | 0.24 (0.86) | 1.45 (4.25) | 2.37 (4.14) | 2.89 (5.94) | 0.9 (2.69) | 0 (0) | 0.45 (2.95) | 0.17 (0.83) |
| Disability pension | 1.88 (6.67) | 1.88 (6.68) | 2.38 (7.9) | 3.9 (17.15) | 4.44 (17.31) | 5.03 (17.65) | 5.54 (17.9) | 5.7 (18.43) | 4.46 (11.4) | 4.03 (8.62) |
| Drugs, PAH-related | 0.02 (0.05) | 0.05 (0.23) | 0.03 (0.08) | 0.21 (0.78) | 1.31 (3.3) | 5.4 (9.96) | 6.35 (12.14) | 8.36 (16.92) | 8.34 (15.45) | 9.12 (18.36) |
| Drugs, non-PAH-related | 0.41 (0.5) | 0.42 (0.51) | 0.4 (0.49) | 0.48 (0.54) | 0.88 (1.33) | 0.96 (1.3) | 0.96 (1.3) | 1.36 (2.65) | 1.88 (6.94) | 1.3 (2.9) |
| **CTEPH, nonoperated** |  |  |  |  |  |  |  |  |  |  |
| Outpatient visits | 1.45 (3.51) | 1.14 (2.26) | 1.04 (1.83) | 1.83 (2.52) | 3.23 (5.44) | 3.28 (3.03) | 2.15 (2.66) | 2.2 (2.23) | 1.9 (2.34) | 1.75 (1.72) |
| Emergency department visits | 0.16 (0.35) | 0.14 (0.3) | 0.21 (0.43) | 0.33 (0.45) | 0.6 (0.69) | 0.34 (0.6) | 0.26 (0.61) | 0.3 (0.76) | 0.16 (0.37) | 0.22 (0.41) |
| Inpatient care | 2.48 (8.13) | 2.83 (9.14) | 4.72 (28.81) | 5.64 (22.39) | 8.58 (11.67) | 7.59 (12.1) | 3.76 (8.27) | 3.63 (7.28) | 1.49 (4.32) | 2.39 (6.32) |
| Institutional care | 0.83 (5.35) | 0.86 (5.08) | 0.53 (4.04) | 0.63 (4.78) | 0.55 (4.05) | 0.07 (0.41) | 0 (0.04) | 0.1 (0.79) | 0.44 (3.51) | 0.37 (2.63) |
| Home care | 0.01 (0.07) | 0.01 (0.05) | 0.01 (0.03) | 0.39 (3.32) | 0.54 (4.38) | 0.37 (2.55) | 0.55 (3.9) | 1.07 (4.97) | 1.22 (6.26) | 0.07 (0.34) |
| Sick leaves | 0.48 (1.98) | 0.46 (2.14) | 0.37 (1.97) | 0.5 (1.92) | 1.03 (2.66) | 1.12 (3.17) | 0.14 (0.72) | 0.22 (0.9) | 0.23 (1.17) | 0.06 (0.46) |
| Disability pension | 3.11 (7.8) | 2.75 (6.55) | 2.75 (6.62) | 2.45 (6) | 2.41 (6.11) | 2.46 (5.84) | 2.13 (5.28) | 1.48 (4.5) | 1.27 (4.38) | 1.34 (4.6) |
| Drugs, PAH-related | 0.03 (0.09) | 0.09 (0.55) | 0.06 (0.16) | 0.17 (0.39) | 1.21 (2.69) | 17.92 (17.6) | 24.48 (19.19) | 25.57 (20.72) | 23.67 (20.38) | 24.25 (23.9) |
| Drugs, non-PAH-related | 1.15 (2.44) | 1.27 (2.74) | 1.17 (2.36) | 1.28 (2.54) | 1.29 (2.69) | 1.39 (3.32) | 1.23 (2.34) | 1.26 (2.52) | 1.05 (2.47) | 0.79 (0.92) |

Data are as mean (SD) number of visits (or days for inpatient, social or sick leave days) per patient per year. CTEPH, Chronic Thromboembolic Pulmonary Hypertension; PAH, Pulmonary Arterial Hypertension.

**Supplementary Table 7. The total 5-year costs of PAH and CTEPH subgroups before and after index date.**

|  | **Before diagnosis,**  **5-year period**,  mean (95%CI) | **After diagnosis,**  **5-year period**,  mean (95%CI) | **Difference**,  mean (95%CI) | **P-value** |
| --- | --- | --- | --- | --- |
| **IPAH and HPAH** |  |  |  |  |
| Total costs | 46 400 (30 800–61 900) | 279 300 (22 1300–337 300) | 233 000 (216 500–249400) | **<0.0001** |
| Outpatient care | 11 400 (4 300–18 500) | 16 400 (13 700–19 100) | 5 000 (-2 100–12 100) | 0.168 |
| Emergency visits | 1 100 (800–1 500) | 1 300 (1 000–1 600) | 200 (-200–600) | 0.307 |
| Inpatient care | 12 000 (8 200–15 800) | 21 700 (16 800–26 600) | 9 700 (5 800–13 500) | **<0.0001** |
| Institutional care | 1 600 (-1 500–4 600) | 11 400 (-13 100–35 900) | 9 900 (6 000–13 700) | **<0.0001** |
| Sick leaves | 3 500 (2 000–4 900) | 4 700 (2 900–6 500) | 1 200 (-300–2 700) | 0.111 |
| Disability pensions | 6 500 (3 000–10 000) | 16 100 (10 100–22 000) | 9 600 (6 000–13 100) | **<0.0001** |
| Drug utilization | 10 200 (5 100–15 300) | 205 400 (156900–253 900) | 195 200 (188 300–202 000) | **<0.0001** |
| Home care | 0 (0–100) | 1 400 (300–2 500) | 1 300 (1 200–1 400) | **<0.0001** |
| **APAH, connective** |  |  |  |  |
| Total costs | 53 100 (39 300–66 900) | 311 100 (246 300–375 900) | 258 000 (242 200–273 700) | **<0.0001** |
| Outpatient care | 13 300 (11 000–15 500) | 22 500 (19 600–25 400) | 9 300 (7 000–11 500) | **<0.0001** |
| Emergency visits | 1 200 (900–1 500) | 2 100 (1 600–2 500) | 800 (500–1 200) | **<0.0001** |
| Inpatient care | 14 900 (6400–23400) | 45 800 (35 200–56 400) | 30 900 (22 300–39 500) | **<0.0001** |
| Institutional care | 0 (0–0) | 300 (200–400) | 300 (300–300) | **<0.0001** |
| Sick leaves | 3 500 (1 800–5 200) | 3 300 (500–6 100) | -200 (-1900–1 500) | 0.798 |
| Disability pensions | 12 600 (6 600–18 600) | 20 900 (8 300–33 400) | 8 300 (2 100–14 400) | **0.009** |
| Drug utilization | 7 600 (4 500–10 700) | 214 400 (154 300–274 600) | 206 800 (198 900–21 4700) | **<0.0001** |
| Home care | 0 (0–100) | 1 500 (200–2 800) | 1 500 (1 300–1 600) | **<0.0001** |
| **APAH, congenital heart disease** |  |  |  |  |
| Total costs | 43 800 (23 900–63 600) | 239 400 (141 800–337 000) | 195 700 (167 500–223 800) | **<0.0001** |
| Outpatient care | 7 400 (2 800–12 000) | 14 100 (10 300–18 000) | 6 700 (2 000–11 400) | **0.007** |
| Emergency visits | 1 200 (600–1 700) | 2 200 (500–4 000) | 1 100 (400–1 700) | **0.002** |
| Inpatient care | 10 100 (5 200–15 100) | 34 400 (15 300–53 400) | 24 300 (18 000–30 500) | **<0.0001** |
| Institutional care | 600 (-600–1 700) | 35 600 (-36 200–107 500) | 35 100 (19 500–50 600) | **0.0001** |
| Sick leaves | 4 200 (1 000–7 300) | 3 800 (600–7 000) | -300 (-3 500–2 900) | 0.827 |
| Disability pensions | 8 800 (300–17 300) | 13 800 (5 000–22 600) | 5 000 (-3 700–13 600) | 0.247 |
| Drug utilization | 11 500 (-600–23 500) | 131 100 (58 700–203 400) | 119 600 (100 400–138 800) | **<0.0001** |
| Home care | 100 (-100–300) | 4 000 (-3 100–11 100) | 3 900 (2 400–5 500) | **<0.0001** |
| **CTEPH, operated** |  |  |  |  |
| Total costs | 47 500 (33 500–61 400) | 125 700 (98 600–152 900) | 78 300 (63 900–92 700) | **<0.0001** |
| Outpatient care | 7 000 (4 200–9 900) | 10 500 (8 500–12 400) | 3 400 (500–6 300) | **0.020** |
| Emergency visits | 1 300 (1 000–1 600) | 1 200 (900–1 600) | -100 (-400–200) | 0.521 |
| Inpatient care | 14 400 (10 500–18 400) | 40 500 (35 500–45 400) | 26 000 (22 000–30 000) | **<0.0001** |
| Institutional care | 200 (-200–700) | 100 (-100–200) | -200 (-600–300) | 0.485 |
| Sick leaves | 5 700 (3 400–8 100) | 4700 (2100–7 200) | -1 100 (-3 500–1 300) | 0.375 |
| Disability pensions | 14 500 (2 100–26 900) | 24300 (5 500–43 100) | 9 800 (-2 800–22 500) | **0.125** |
| Drug utilization | 4 200 (3 100–5 300) | 44 100 (29 000–59 200) | 39 900 (37 600–42 100) | **<0.0001** |
| Home care | 3 (0–6) | 286 (65–507) | 283 (254–312) | **<0.0001** |
| **CTEPH, nonoperated** |  |  |  |  |
| Total costs | 62 400 (44 000–80 900) | 170 400 (149100–191600) | 107900 (89400–126500) | **<0.0001** |
| Outpatient care | 87 00 (6 800–10 600) | 10 800 (9 200–12 400) | 2 100 (200–4 000) | **0.033** |
| Emergency visits | 1 400 (1 200–1 700) | 1 300 (1 000–1 600) | -200 (-400–100) | 0.238 |
| Inpatient care | 24 200 (12 300–36 200) | 22 300 (18 800–25 800) | -1900 (-13 900–10 000) | 0.750 |
| Institutional care | 3 400 (-500–7 300) | 900 (-700–2 600) | -2 500 (-6 400–1 400) | 0.214 |
| Sick leaves | 2 800 (1 500–4 200) | 1 800 (1 100–2 500) | -1 100 (-2 400–300) | 0.131 |
| Disability pensions | 13 500 (7 800–19 100) | 8 400 (3 900–13 000) | -5 000 (-10 700–600) | 0.080 |
| Drug utilization | 7 700 (5 400–10 100) | 121 200 (102 700–139 700) | 113 500 (110 600–116 400) | **<0.0001** |
| Home care | 900 (-800–2600) | 3 200 (800–5 600) | 2 300 (900–3 700) | **0.002** |

Data are shown as mean (95% confidence interval), per patient, EUR. Zhao and Tian (ZT) estimator was used to adjust for censoring.
